# Severe Intraperitoneal Haemorrhage following Suprapubic Catheter Insertion in a Patient Treated with Iloprost

**DOI:** 10.1155/2013/724685

**Published:** 2013-08-22

**Authors:** R. A. J. Spence, A. Thwaini, Aidan O'Brien

**Affiliations:** Department of Urology, Craigavon Area Hospital, 68 Lurgan Road, Portadown BT63 5QQ, UK

## Abstract

Suprapubic catheter (SPC) insertion is a common urological procedure, performed both in the elective and emergency settings. The authors present an unusual case of severe intraperitoneal bleeding following the insertion of an SPC under direct vision, where the use of prostacyclin analogue may have been a contributing factor.

## 1. Introduction

Prostacyclin analogue (Iloprost) is used in a variety of clinical conditions, for example, pulmonary hypertension and Raynaud's phenomenon. We report a case of a 42-year-old female who had an elective insertion of SPC. She had an intravenous infusion of Iloprost earlier that day. Following catheter insertion, she developed severe postoperative bleeding requiring laparotomy. To our knowledge this is the first case reported in the literature with a complication related to prostacyclin infusion.

## 2. Case Report

A 42-year-old female patient with advanced multiple sclerosis (MS) for the last 13 years was admitted electively under the medical team with a new diagnosis of Reynaud's phenomenon. During her admission she expressed an interest in having an SPC inserted instead of her long term urethral catheter.

She had been recently commenced on daily intravenous infusion of prostacyclin analogue (Iloprost) to treat her Reynaud's phenomenon. After counselling, the patient was booked for cystoscopy (under general anaesthesia) and SPC insertion. She had finished her fifth day of iloprost at 12 pm. She had her procedure performed at 4 pm with no intraoperative issues. 

Three hours later, whilst back on the ward, the patient developed severe generalised abdominal pain and became unstable with hypotension and tachycardia. Her haemoglobin dropped from 12 gm/L to 8 gm/L.

**Figure 1:**
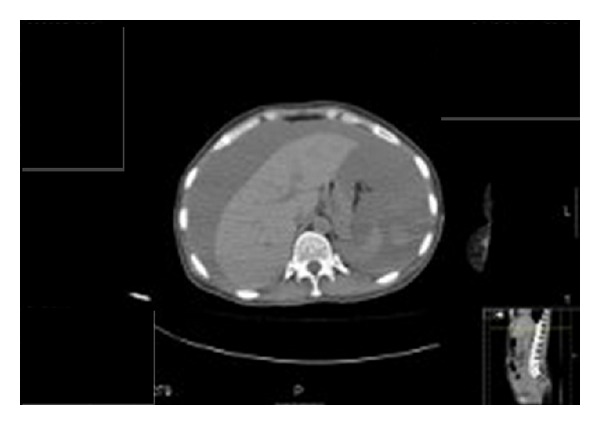
CT scan demonstrating significant intraperitoneal bleeding.

She was stabilised with intravenous plasma expanders and packed red blood cells. An emergency computed tomography (CT) scan confirmed intraabdominal bleeding (Figure 1).

She was immediately taken to the theatre for an emergency laparotomy. There was approximately 3 litres of blood within her abdomen, with intra-peritoneal blood clots visible. The catheter was found to be in the urinary bladde, but had followed an intraperitoneal path, with a small haematoma on the peritoneal surface of the bladder. No active bleeding was found, and it was assumed that the bleeding was from the dome of the bladder.

She recovered well from the operation and was discharged in a good health.

## 3. Discussion

Insertion of SPC is a common procedure in urology. Despite the fact that this procedure is relatively safe, it is not without risks, and there is a paucity of evidence published in the literature about its potential complications. The incidence of such complications is usually minimised by performing the procedure under direct vision of the cystoscope. However it is not completely safe, even in the experienced hands [1].

There have been several complications reported from SPC insertion, under direct vision, yet excessive bleeding necessitating operative exploration has not been reported as far as we are aware.

In our case, the patient has been commenced on Iloprost for Reynaud's phenomenon. Iloprost has been used for a variety of conditions, with one example being limb ischaemia. It is thought that Iloprost has mild yet sustained inhibition of platelets aggregation, hence it is useful in peripheral vascular disease [2].

In addition, it has been used to preserve the platelets in extracorporeal circulation. This is achieved via inhibition of platelets aggregation and hence protects platelets from consumption [3].

At time of laparotomy there was approximately 3 litres of intraperitoneal blood, with only small amounts of blood clot. The SPC, in fact, was draining clear urine despite the massive intraperitoneal bleeding. The coagulation profile was normal prior to the SPC insertion and remained within normal values prior to laparotomy. However, selective platelets function tests were not performed as there was no suspicion of platelets dysfunction at the time. Intraperitoneal placement of the SPC may have been a further factor in the continuing bleeding. This might have abolished the tamponading effect of the extraperitoneal placement of the SPC.

## 4. Conclusions

The insertion of SPC should not be underestimated as it carries significant morbidity, and reported mortality. In addition, the effect of Iloprost on the platelets function is to be considered prior to any surgical intervention, with contemplation of functional platelets studies prior to any surgical undertaking.
